# Physiological performance of transplastomic tobacco plants overexpressing aquaporin AQP1 in chloroplast membranes

**DOI:** 10.1093/jxb/ery148

**Published:** 2018-04-18

**Authors:** Alicia Fernández-San Millán, Iker Aranjuelo, Cyril Douthe, Miquel Nadal, María Ancín, Luis Larraya, Inmaculada Farran, Jaume Flexas, Jon Veramendi

**Affiliations:** 1 Instituto de Agrobiotecnología (Universidad Pública de Navarra-CSIC), Departamento de Producción Agraria, Campus Arrosadía, Pamplona, Spain; 2 Research Group on Plant Biology under Mediterranean Conditions, Departament de Biologia, Universitat de les Illes Balears, Carretera de Valldemossa, Palma de Mallorca, Illes Balears, Spain

**Keywords:** Aquaporin, chloroplast envelope, CO_2_ permeability, plastid transformation, protein targeting, tobacco

## Abstract

The leaf mesophyll CO_2_ conductance and the concentration of CO_2_ within the chloroplast are major factors affecting photosynthetic performance. Previous studies have shown that the aquaporin NtAQP1 (which localizes to the plasma membrane and chloroplast inner envelope membrane) is involved in CO_2_ permeability in the chloroplast. Levels of NtAQP1 in plants genetically engineered to overexpress the protein correlated positively with leaf mesophyll CO_2_ conductance and photosynthetic rate. In these studies, the nuclear transformation method used led to changes in NtAQP1 levels in the plasma membrane and the chloroplast inner envelope membrane. In the present work, NtAQP1 levels were increased up to 16-fold in the chloroplast membranes alone by the overexpression of *NtAQP1* from the plastid genome. Despite the high NtAQP1 levels achieved, transplastomic plants showed lower photosynthetic rates than wild-type plants. This result was associated with lower Rubisco maximum carboxylation rate and ribulose 1,5-bisphosphate regeneration. Transplastomic plants showed reduced mesophyll CO_2_ conductance but no changes in chloroplast CO_2_ concentration. The absence of differences in chloroplast CO_2_ concentration was associated with the lower CO_2_ fixation activity of the transplastomic plants. These findings suggest that non-functional pores of recombinant NtAQP1 may be produced in the chloroplast inner envelope membrane.

## Teaser

Overexpression of *NtAQP1* from the tobacco plastid genome resulted in impaired photosynthetic performance of transplastomic tobacco plants.

## Introduction

It is predicted that future increases in the human population will require a 30% increase in crop yield rates (Edgerton, 2009). Improving the photosynthetic performance of crops is one way in which plant production might be increased (Parry *et al.*, 2011; Reynolds *et al.*, 2011; Parry *et al.*, 2013; Flexas *et al.*, 2013), and a number of strategies have been identified that, either individually or in combination, might achieve this (Long *et al.*, 2006; Flexas *et al.*, 2006; Parry *et al.*, 2013; Flexas *et al.*, 2012). Photosynthetic performance is affected by two major factors: the concentration of CO_2_ within the chloroplast and the efficiency of the carboxylation biochemistry. Availability of CO_2_ at the carboxylation site in the chloroplast can be limited by its diffusion into the substomatal cavities, referred to as stomatal conductance (*g*_s_), and by the conductance of CO_2_ from the substomatal cavity to the chloroplast, referred to as mesophyll conductance (*g*_m_). Classically, *g*_m_ has been described not to limit photosynthesis, and the CO_2_ concentration was thought to be similar in the substomatal cavity (C_i_) and in the chloroplast stroma (C_c_). However, over the past decade, a number of studies (Flexas *et al.*, 2006; Scafaro *et al.*, 2011; Kaldenhoff, 2012; Evans and von Caemmerer, 2013; Flexas *et al.*, 2013) have shown that *g*_m_ has a major influence on CO_2_ diffusion into the chloroplast, with a consequent impact on the photosynthetic rate. At the cellular level, atmospheric CO_2_ has to pass through the cell wall and three membranes (the plasma membrane and the two membranes of the chloroplast envelope) to reach the chloroplast stroma. The CO_2_ permeability of the chloroplast envelope is low, probably due to its relatively large protein content (Priestley and Woolhouse, 1980); indeed, it was estimated that it may account for almost half of the internal leaf resistance to CO_2_ (Uehlein *et al.*, 2008). As a result, under light-saturated conditions, photosynthesis is limited by the availability of CO_2_ within the chloroplast. Other studies have shown that the *g*_m_ can change quickly in response to varying environmental conditions, such as leaf temperature (Bernacchi *et al.*, 2002), water stress (Galmés *et al.*, 2007), blue light (Loreto *et al.*, 2009), and the external CO_2_ concentration (Flexas *et al.*, 2007*a*). This rapid modification of *g*_m_ points to the existence of additional components, some of them probably proteins, controlling the conductance of the mesophyll to CO_2_ diffusion. Proteins forming pore-like structures, such as aquaporins (AQPs), might help explain how these rapid variations in *g*_m_ occur.

AQPs are small proteins that increase the permeability of cell membranes to water and certain small, neutral molecules, including CO_2_ (Maurel *et al.*, 2008; Gomes *et al.*, 2009; Chaumont and Tyerman, 2014; Kaldenhoff *et al.*, 2014; Groszmann *et al.*, 2017). AQPs were discovered for the first time in plants in the vacuolar tonoplast of Arabidopsis (Maurel *et al.*, 1993), and are present in the whole plant kingdom. AQPs are located in the plasma membrane and also in most of the intracellular membranes. Many isoforms of AQPs exist, which can be classified according to their sequence homologies and subcellular localization. The plasma membrane intrinsic protein (PIP) class includes isoforms that are most abundant in the plasma membrane. This class can be subdivided into subclasses PIP1 and PIP2 according to sequence similarity. Investigations on the mesophyll cells of tobacco leaves have shown that the plasma membrane protein NtAQP1 (a PIP1 member) facilitates CO_2_ transport, and that it has important functions in photosynthesis and stomatal opening (Uehlein *et al.*, 2003). Further studies revealed a dual localization of NtAQP1 in the plasma membrane and the inner envelope membrane (IEM) of the chloroplast (Uehlein *et al.*, 2008). A mutation in the *Arabidopsis thaliana AtPIP1;2* gene was found to be associated with reduced *g*_m_ and a reduction in the rate of photosynthesis (Heckwolf *et al.*, 2011). Genetic engineering to modify NtAQP1 expression levels confirmed these results, revealing a function for NtAQP1 in CO_2_ conductance. Antisense or RNA interference-mediated downregulation of *NtAQP1* resulted in a reduction of IEM CO_2_ permeability, C_c_ values, and photosynthetic performance (Uehlein *et al.*, 2003; Flexas *et al.*, 2006; Uehlein *et al.*, 2008). Overexpression of *NtAQP1* in tobacco and Arabidopsis, however, increased chloroplast membrane CO_2_ permeability, the rate of photosynthesis, and plant growth (Aharon *et al.*, 2003; Uehlein *et al.*, 2003; Flexas *et al.*, 2006). A similar positive effect on CO_2_ permeability, plus an increase in leaf net photosynthesis, was observed in tomato and rice plants overexpressing AQP (Hanba *et al.*, 2004; Sade *et al.*, 2010). It was eventually suggested that the function of NtAQP1 might depend on its localization in the cell, and that it might provide a water channel in the plasma membrane and a CO_2_ channel in the chloroplast envelope (Uehlein *et al.*, 2008).

In the present study, the hypothesis that higher levels of AQPs in the chloroplast would increase CO_2_ transport and the rate of photosynthesis was tested by overexpressing *NtAQP1* from the chloroplast genome of tobacco. Compared with nuclear transformation, plastid transformation provides the advantage of high transgene expression levels (Bock, 2015). In addition, the recombinant protein is confined to the chloroplast, eliminating the effect of AQP1 modification in the plasma membrane. Therefore, the main objective of the present study was to evaluate the role of *NtAQP1* overexpression specifically in the chloroplast membranes on CO_2_ permeability and photosynthetic performance.

## Materials and methods

### Production of plants overexpressing NtAQP1 in the chloroplast

Total RNA from *Nicotiana tabacum* L. (cv. Petite Havana SR1) leaves was extracted using the Ultraspec RNA kit (Biotecx Laboratories, Houston, TX, USA), and cDNA was synthesized using the SuperScript III system (Invitrogen, Carlsbad, CA, USA). The *NtAQP1* gene (GenBank Accession AJ001416) was amplified by PCR with the primers NTAQP1for: AAGCTTTTGCAAGTATATT TTCCATGGCAGAAAACAAAGAA GAAGATGTTAAGCTCGG and NTAQP1rev: GCGGCCGCTTAA GACGACTTG TGGAATGGAATGGCTCTG. The full-length cDNA was then cloned into the pGEMTeasy vector (Promega, Madison, WI, USA) and sequenced. The tobacco *NtAQP1* gene was subsequently cloned into the pAF chloroplast transformation vector (Fernández-San Millán *et al.*, 2008) under the control of the *psbA* promoter and 5ʹ-untranslated region, to obtain the expression vector pAF-AQP1. The Tic40 transit peptide sequence (240 bp) was amplified by PCR using cDNA from *A. thaliana* using the primers AtTic40TPfor: CCATGGAGAACCTTACCCTAGTTTC and AtTic40TPrev: GCGGCCGCAAGCTTTGCTTCTCTGTTTC. It was then fused with *NtAQP1* at an *Nco*I restriction site to produce the expression vector pAF-TicAQP1.

*N. tabacum* L. (cv. Petite Havana SR1) was also used in plastid transformations. The PDS-1000/He biolistic system (Bio-Rad, Hercules, CA, USA) was used for the integration of transgenes as previously described (Daniell, 1997). The *aadA* gene, conferring resistance to spectinomycin, was used as a selectable marker gene. Two rounds of selection and shoot development on RMOP medium containing 500 mg/l spectinomycin were performed. The transplastomic plants produced were named AQP1 and TicAQP1.

### Southern and northern blotting

Southern and northern blotting experiments were performed as previously described (Sanz-Barrio *et al.*, 2013). For Southern blotting, the flanking sequence P1 probe generated by PCR was used. After Southern blot confirmation of the T_0_ generation, selected plants were transplanted into pots and grown in the greenhouse for seed production. T_1_ plants were used in further experiments. Northern blotting was performed using the AQP1-specific P2 probe (515 bp) obtained by *Nco*I digestion of *AQP1*.

### Protein extraction, separation, and western blotting

Leaf samples (100 mg) from transformed and untransformed 70-day-old plants were ground in liquid nitrogen, homogenized in 300 μl of 2× Laemmli buffer (0.5 M Tris–HCl, pH 6.5, 4% SDS, 20% glycerol, and 10% β-mercaptoethanol) and heated at 95 °C for 5 min. After 5 min of centrifugation at 20000 *g*, the supernatant was deemed to represent the total protein (TP) content. TP was quantified using the RC-DC protein assay (Bio-Rad) with BSA as a standard. Proteins were separated by SDS-PAGE on 12% polyacrylamide gels and transferred to a polyvinylidene fluoride (PVDF) membrane for immunoblotting. The primary antibodies used were anti-PIP1 (Agrisera, Vännäs, Sweden) and anti-NtAQP1 (kindly provided by R. Kaldenhoff) (dilution 1:3000). Peroxidase-conjugated goat anti-rabbit or anti-chicken immunoglobulin G (Sigma-Aldrich, St Louis, MO, USA) (both at a dilution of 1:3000) were used as secondary antibodies with the anti-PIP1 and anti-NtAQP1 primary antibodies, respectively. Detection was performed using the chemiluminescence ECL western blotting system (GE Healthcare, Fairfield, CT, USA).

Relative quantification of NtAQP1 monomers and oligomers was performed by comparing dilution series of TP from wild-type (WT) plants and both types of transplastomic plant (three replicates were analysed). For each line, adequate amounts were loaded on to an SDS-PAGE gel, electrophoretically separated, and then analysed by western blotting. Immunoblots were quantified using GeneTools Analyzer software (SynGene, Cambridge, UK).

### Plasma membrane isolation

Plasma membranes from 50-day-old WT and transplastomic plants grown in a growth chamber [16 h light/8 h dark; 200 µmol m^−2^ s^−1^ photosynthetic photon flux density (PPFD) and a day/night temperature regime of 28 °C/25 °C] were obtained as previously described (Santoni, 2007).

### Chloroplast isolation, fractionation, and immunoblotting

For chloroplast isolation, leaves from 50-day-old tobacco plants were cut into 1–3 cm^2^ pieces and homogenized in a blender. The isolation buffer (330 mM sorbitol, 20 mM MOPS, 13 mM Tris, 3 mM MgCl_2_, 0.1% BSA) was six times (v/w) the fresh mass weight of the leaf samples. The homogenate was passed through a filter mesh and centrifuged for 5 min at 1000 *g* and 4 °C. The pellet fraction was resuspended in isolation buffer and chloroplasts were isolated by 80–40% Percoll gradient fractionation after centrifugation for 10 min at 7700 *g* and 4 °C. Isolated chloroplasts were washed in 3 volumes of washing buffer (330 mM sorbitol, 50 mM HEPES/KOH, pH 7.6, 3 mM MgCl_2_).

For fractionation, the chloroplasts were lysed by freeze-thawing in hypotonic TE buffer [10 mM Tris, 2 mM EDTA, pH 7.5, including a cocktail of protease inhibitors from Roche (Mannheim, Germany)]. Stroma, envelopes, and thylakoids were separated by using discontinuous sucrose gradients (0.93/0.6/0.3 M) after 2 h of centrifugation at 20000 rpm in a swing-out rotor. Stroma was collected from the upper fractions and one volume of extracts was combined with one volume of 2× Laemmli buffer. The thylakoid membranes sedimented out and were resuspended in 10 mM TE buffer, to which one volume of 2× Laemmli buffer was added. The chloroplast envelopes were collected at the interface between 0.9 and 0.6 M sucrose. The envelope proteins were concentrated by methanol/chloroform extraction (Ferro *et al.*, 2002) and resuspended in 10 mM TE buffer with one volume of 2× Laemmli buffer added. All three fractions were heated for 1 h at 37 °C. Protein concentrations were determined by the Bradford method. All samples were resolved by 9% SDS-PAGE and transferred to PVDF membranes for western blotting. Antisera to ADP-glucose pyrophosphorylase (AGPase), LHC chlorophyll a/b binding protein 1 (Lhcb1), and Tic40 (Agrisera) proteins were used at dilutions of 1:1000.

### Plants used for gas exchange

Plants were grown in 2 litre pots (organic soil/perlite, 70/30 v/v) in two places, Pamplona and Mallorca (Spain). In Pamplona, plants were grown in a greenhouse at 24–28 °C and relative humidity ~40%. Plants were watered with water by drip irrigation and twice a week with 50% diluted Hoagland’s solution. In Mallorca, plants were grown in a growth chamber at PPFD ~350 µmol m^−2^ s^−1^ at the top of the plants, daily temperature of 24–26 °C, relative humidity ~40%, and watered twice a week with water and once a week with 50% diluted Hoagland’s solution.

### Gas exchange and chlorophyll fluorescence analyses

Gas exchange measurements were performed with a calibrated Li-6400 XT portable gas analyser (Licor, Lincoln, NE, USA) equipped with the 2 cm^2^ Li-6400-40 Leaf Chamber Fluorometer. Determinations were conducted in apical fully developed leaves. Three independent experiments (two in Pamplona and one in Mallorca) were performed. For the measurements made in Pamplona, plants were transferred to a growth chamber with similar environmental conditions to those of the Mallorca site. For each plant, the same procedure was followed: first, stabilization until a steady state of stomatal conductance was reached (typically ~20–30 minutes) in ambient conditions (CO_2_ concentration=400 µmol mol^−1^, 1500 PPFD, and 25 °C). After stabilization, the A_N_/C_i_ curve was performed by changing the concentration of CO_2_ entering the leaf chamber with, the following steps: 400, 300, 250, 200, 150, 100, 50, 400, 400, 500, 600, 700, 800, 1000, 1200 and 1500 µmol mol^−1^, with typically 2–3 minutes between each step. Each A_N_/C_i_ curve was corrected for leaks by following the protocol described by Flexas *et al.*, (2007*b*). In all three experiments, the results of net CO_2_ assimilation and stomatal conductance were very consistent (data not shown). In the third experiment, performed in Mallorca, chlorophyll fluorescence was measured together with gas exchange to estimate the mesophyll conductance to CO_2_. Therefore, all the results shown in the present paper correspond to this latter experiment.

After performing the A_N_/C_i_ curve, the leaf was kept in the chamber and N_2_ from a tank (Air Liquide) was piped into the Li-6400 inlet to remove O_2_ from the entering air in the leaf chamber, to allow measurements to be made in non-photorespiratory conditions (Valentini *et al.*, 1995). We then performed a light curve at ambient CO_2_ concentration (400 µmol mol^−1^) with the following PPFD steps: 1500, 2000, 1750, 1500, 1250, 1000, 800, 550, 300, 150, 100, 75, 50, 25 and 0 µmol m^−2^ s^−1^. These measurements were used to estimate the product of leaf absorption (α) and the partitioning of absorbed quanta between photosystems I and II (β) (see Valentini *et al.*, 1995 and Pons *et al.*, 2009 for details). We used only the first points of the curve, with PPFD >400 µmol m^−2^ s^−1^, to estimate α*β (Martins *et al.*, 2013), avoiding non-linearity of ΦPSII versus ΦCO_2_ due to changes/higher influence of leaf respiration at low PPFD. Values of (α*β) were 0.36 ± 0.04 (TicAQP1), 0.35 ± 0.03 (AQP1), and 0.38 ± 0.03 (WT), with no significant differences between genotypes (see Supplementary Fig. S1 at *JXB* online). Night respiration rate (R_d_) was estimated by measuring leaf gas exchange in darkness, 1 h after the lights of the growing chamber were turned off (night).

Mesophyll conductance (*g*_*m*_) was estimated by the method developed by Harley *et al.* (1992), as follows:

$$g_{m}={\text{A}}_{N}/({\text{C}}_{i}-\{\Gamma *\left[ETR+\text{8}\left({\text{A}}_{N}+{\text{R}}_{l}\right)\right]/\left[\text{ETR}–\text{4}\left({\text{A}}_{N}+{\text{R}}_{l}\right)\right]\})$$

where A_N_ is the net CO_2_ assimilation rate, C_i_ is the CO_2_ concentration in the substomatal cavity, Γ* is the CO_2_ compensation point in the absence of R_d_ (assumed to be 40 µmol mol^−1^, from Walker *et al.*, 2013), R_l_ is the respiration rate in light (estimated as R_d_/2), and *ETR* is the electron transport rate, estimated as follows:

ETR=α×β×ΦPSII×PPFD

where ΦPSII is the yield of photosystem 2. ΦPSII was estimated using the ‘Multiphase Flash’ method described by Loriaux *et al.* (2013).

### Discrimination against ^13^CO_2_

The ^13^C isotope discrimination (Δ, ‰) was calculated as:

$$\Delta =\frac{\delta^{\text{13}}{\text{C}}_{\text{atm}}-\delta^{\text{13}}{\text{C}}_{\text{sample}}}{\delta^{\text{13}}{\text{C}}_{\text{sample}}+1}$$

where δ^13^C_atm_ is the carbon isotope composition in atmospheric CO_2_ in the greenhouse (–10.8‰) and δ^13^C_sample_ is the carbon isotope composition of leaf total organic matter (TOM).

The ^13^C/^12^C ratio (R) in plant material was expressed in δ notation (δ^13^C) with respect to Vienna Pee Dee Belemnite calcium carbonate (V-PDB), and measured with an analytical precision of 0.1‰:

$$\delta^{\text{13}}\text{C=}\left(\frac{{\text{R}}_{\text{sample}}}{{\text{R}}_{\text{standard}}}\right)-1$$

δ^13^C accuracy was monitored using international secondary standards of known ^13^C/^12^C ratios (IAEA-CH7 polyethylene foil, IAEA-CH6 sucrose, and USGS-40 glutamic acid, IAEA, Austria).

TOM and gas δ^13^C determinations were conducted at the Serveis Cientifico-Tecnics of the University of Barcelona. For TOM analyses, 1 mg of dry ground leaf material was analysed using an elemental analyser (EA1108, Series 1, Carbo Erba Instrumentazione, Milan, Italy) coupled to an isotope ratio mass spectrometer (Delta C, Finnigan MAT, Bremen, Germany) operating in continuous flow mode. Air δ^13^C samples were analysed by gas chromatography (Agilent 6890 Gas Chromatograph, Agilent Technologies, Spain) coupled to an isotope ratio mass spectrometer Deltaplus via a GC-C Combustion III interphase (ThermoFinnigan, Thermo, Barcelona, Spain).

### Determination of Rubisco, starch, and chlorophyll contents

Samples from the same leaves used for gas exchange and chlorophyll fluorescence measurements were analysed for their Rubisco, starch, and chlorophyll contents. Three leaf discs (2.1 cm^2^) per plant from WT, AQP1, and TicAQP1 plants were frozen in liquid nitrogen and ground in a Mikro-dismembrator (Braun, Melsungen, Germany). The volume of the extraction buffer (phosphate buffer, pH 7.0, 100 mM) was three times the fresh weight (v/w) of the powdered leaf sample obtained from the three leaf discs. Samples were mixed in a vortex and, after 15 min on ice, centrifuged for 5 min at 20000 *g* at 4 °C. Protein fractions recovered from the supernatants were quantified by the Bradford method. For the separation of these proteins, one volume of the protein fraction was combined with one volume of 2× Laemmli buffer, boiled for 5 min, and then centrifuged at 20000 *g* for 5 min. Samples (15 μg) of the proteins in these supernatants were separated by SDS-PAGE (10%) and the gels were stained with Coomassie brilliant blue G-250. The Rubisco levels of the transplastomic plants were compared with those of the WT plants using GeneTools Analyzer software (SynGene).

Starch was determined using an amyloglucosidase-based test kit (R-Biopharm AG, Darmstadt, Germany).

The leaf chlorophyll contents of the transplastomic and WT plants [measured with a SPAD 502 chlorophyll meter (Minolta Optics Inc, Tokyo, Japan)] were recorded in the same leaves used for photosynthetic rate measurements.

Leaf area of flowering plants grown in a growth chamber was determined after scanning with ImageJ.

### Electron microscopy

Samples from the same leaves used to measure the photosynthetic rate were fixed in Karnovsky fixative (4% formaldehyde and 5% glutaraldehyde in 0.025 M cacodylate buffer, pH 6.7) by vacuum infiltration and further prepared for examination by transmission electron microscopy at the Microscopy Service of the University of Navarre, Spain.

### Statistical analysis

One-way ANOVA was used to analyse differences in the measured variables between the control and transplastomic plants. Differences among means were analysed using the Tukey test (*P*<0.05). All calculations were performed using SPSS 10.0 software.

## Results

### Generation of tobacco transplastomic plants and determination of homoplasmy

**Fig. 1.**
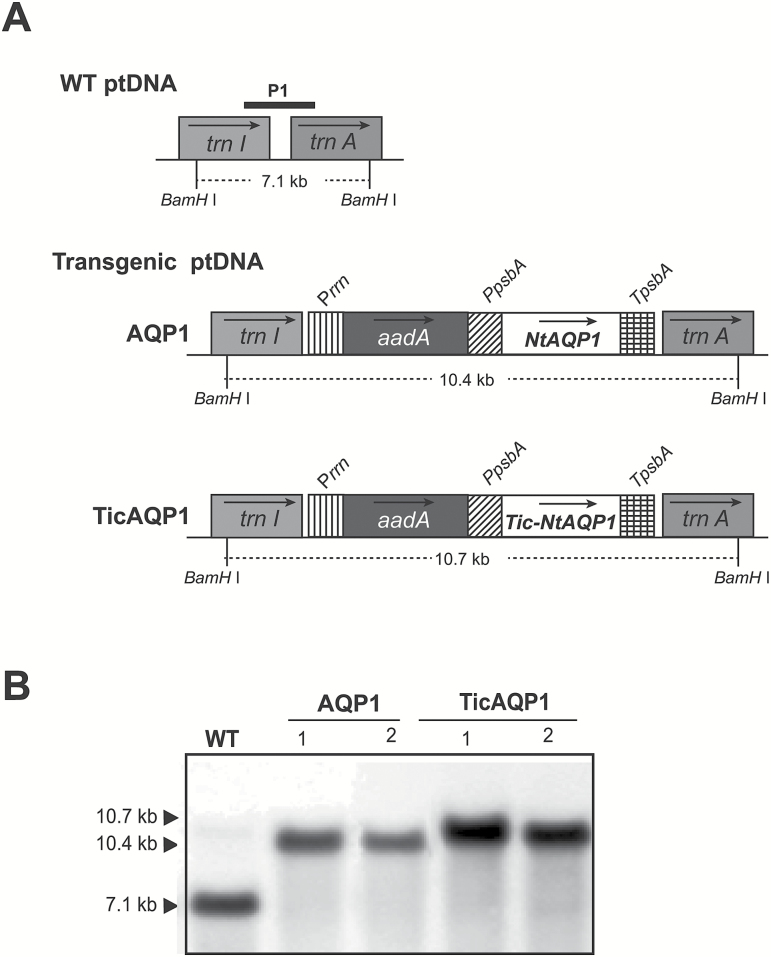
Integration of *Nicotiana tabacum AQP1* into the tobacco chloroplast genome. (A) Map of the wild-type (WT) and *AQP1*-transformed plastid (pt) genomes. The transgenes were targeted to the intergenic region between *trnI* and *trnA*. The selectable marker gene *aadA* (encoding aminoglycoside 3ʹ-adenylyltransferase) was driven by the 16S ribosomal RNA operon promoter (*Prrn*). *AQP1* was driven by the *psbA* promoter and 5ʹ-untranslated region (*PpsbA*). Arrows within boxes show the direction of transcription. Numbers below each ptDNA indicate the predicted size of hybridizing fragments when total DNA was digested with *Bam*HI. A 0.8 kb fragment of the targeting region for homologous recombination was used as a probe (P1) for Southern blot analysis. *TpsbA*, 3ʹ-untranslated region of the *psbA* gene. (B) Southern blot analysis of two independent lines (1 and 2) for each transformation cassette.

Tobacco plants expressing *NtAQP1* from the plastid genome were obtained by biolistic bombardment of the leaves with the engineered pAF vector (Fernández-San Millán *et al.*, 2008), which inserted the transgenes between the *trnI* and *trnA* regions of the plastid genome (Fig. 1A). Two different transformation vectors were designed, both with the transgene controlled by the promoter and the 5ʹ-untranslated region of the *psbA* gene. The pAF-AQP1 vector included the full coding sequence of *NtAQP1*. In the pAF-TicAQP1 vector, the 76 amino acid sequence transit peptide of *A. thaliana* Tic40 protein was fused to the N-terminus of NtAQP1. Two independent transplastomic lines for each construction, developed after two rounds of selection on spectinomycin, were analysed by Southern blotting (Fig. 1B). As predicted for the correct homologous recombination of the transgenes, the flanking region P1 probe hybridized to a 10.4 or 10.7 kb *Bam*HI DNA fragment in the AQP1 and TicAQP1 transplastomic plants, respectively. A 7.1 kb band was detected only in the WT plants, indicating that all four lines were homoplasmic.

### Expression of aquaporin in the chloroplast

**Fig. 2.**
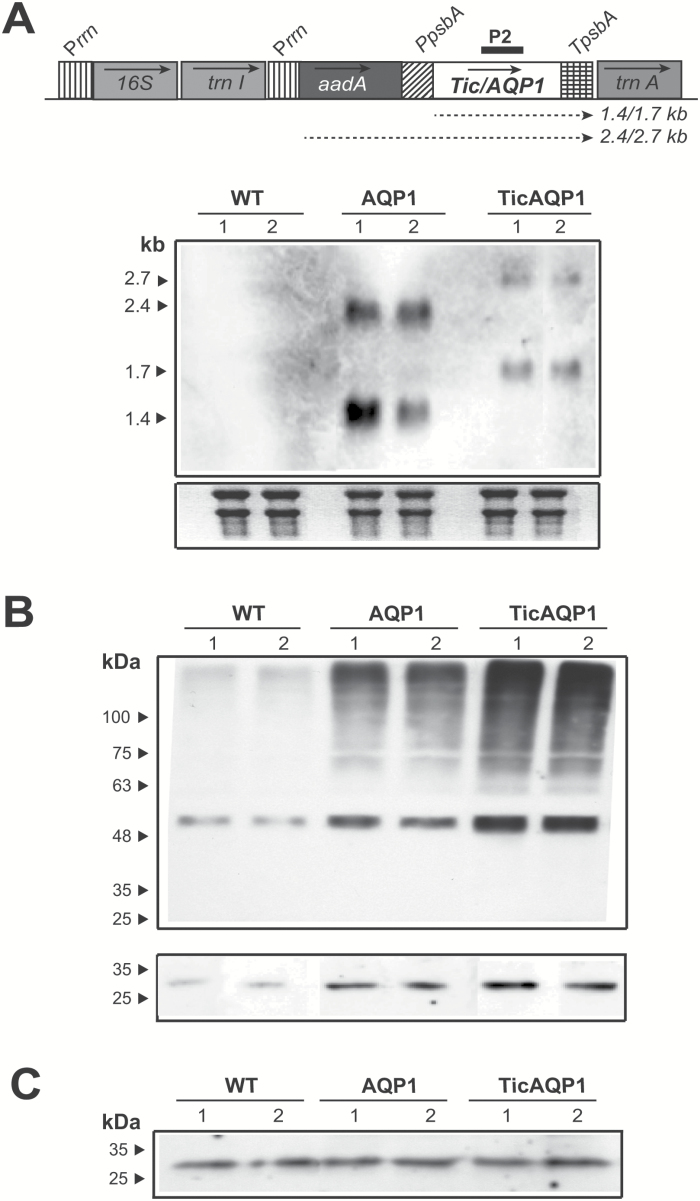
Analysis of *AQP1* expression in wild-type (WT) and AQP1 and TicAQP1 transplastomic plants. (A) Northern blot analysis of leaf samples. The expected transcript sizes of the mono- and dicistrons originating from different promoters are indicated below the map of the transformed plastid genome. The 515 bp *AQP1* sequence (P2) was used as a probe. A 10 μg aliquot of total RNA was loaded per well. Ethidium bromide-stained rRNA was used to assess loading. (B) Western blot analysis of total protein from leaf samples (two independent lines for each construction). The lower panel was overexposed to show the 30 kDa AQP1 monomer, which was not detected in the upper panel. A 30 μg aliquot of protein was loaded per well. (C) Western blot analysis of proteins extracted from the plasma membrane. A 3 μg aliquot of protein was loaded per well. The positions and sizes of molecular weight protein standards are indicated. The blots in B and C were detected using anti-NtAQP1 as the primary antibody.

Analysis at the transcriptional level in the transplastomic plants revealed transcripts of the expected size in both cases (Fig. 2A). Monocistrons of 1.4 and 1.7 kb derived from the *psbA* promoter were detected in the AQP1 and TicAQP1 plants, respectively. Dicistrons transcribed from the upstream *rrn* promoter were also present. A greater abundance of transcripts was observed in the AQP1 plants than in the TicAQP1 plants, probably owing to the TicAQP1 transcripts being less stable. The endogenous AQP1 mRNA was below the detection limit.

The overexpression of AQP1 protein in the transformed chloroplasts was confirmed by immunoblotting (Fig. 2B). A faint 30 kDa band of the expected size for the AQP1 protein monomer was observed in the WT plants. A stronger signal of the same electrophoretic mobility was detected in the AQP1 and TicAQP1 plants. Thus, the TicAQP1 recombinant protein was correctly processed in the stroma of the chloroplast following cleavage of the Tic40 transit peptide. Higher molecular weight signals were mainly present in transplastomic plants, indicating the presence of abundant oligomeric structures despite the denaturing conditions used during sample preparation and electrophoresis. It is also possible that the increased AQP1 protein production or inadequate post-translational modifications caused misfolding or the formation of non-specific aggregates with other proteins that resulted in abnormal migration patterns in the gel. The putative non-specific protein aggregates of high molecular weight could indicate that only a proportion of the recombinant protein equates to functional AQP1 complexes. Relative AQP1 protein levels were estimated by densitometry of different protein extract dilutions in western blots, analysing both monomeric and multimeric signals. The expression level of AQP1 protein in the AQP1 and TicAQP1 plants was approximately 10-fold and 16-fold greater, respectively, than in the WT plants.

As expected, analysis of purified plasma membranes by immunoblotting indicated that the AQP1 protein levels in the WT and transplastomic plants were similar (Fig. 2C), confirming that the AQP1 protein synthesized in the chloroplast was not exported out of the chloroplast.

### NtAQP1 was mainly localized to the chloroplast envelope

**Fig. 3.**
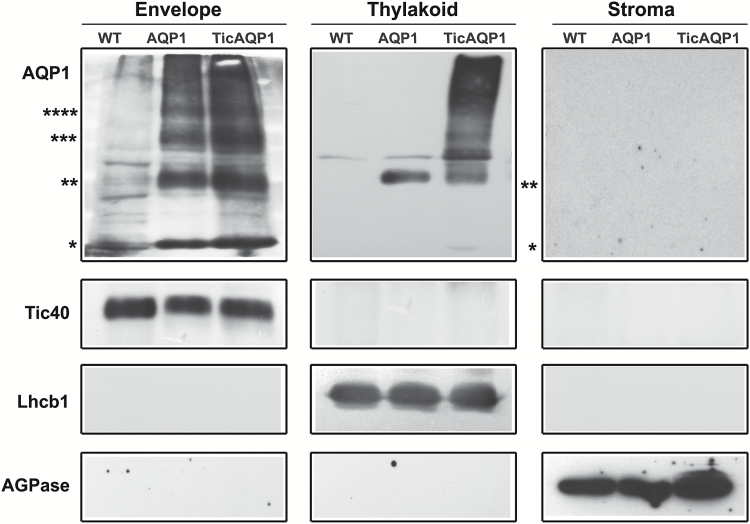
Localization of AQP1 in the thylakoid and envelope membranes. Envelope, thylakoid, and stroma fractions were isolated from wild-type (WT), AQP1, and TicAQP1 leaves, and separated by SDS-PAGE. Samples of 2, 20, and 30 μg of protein from the envelope, thylakoid, and stroma, respectively, were loaded per well. Representative western blots performed with antibodies to AQP1, the inner-membrane Tic40 protein, the thylakoid membrane-specific LHC chlorophyll a/b binding protein 1 (Lhcb1), and the stroma-specific ADP-glucose pyrophosphorylase (AGPase) are shown. Asterisks indicate the positions of monomer (*), dimer (**), trimer (***), and tetramer (****) AQP1.

The distribution of AQP1 in the chloroplast of the AQP1 and TicAQP1 transplastomic plants was examined by chloroplast purification and suborganellar fractionation followed by immunoblotting (Fig. 3). The equal loading of gels for each fraction and the purity of the fractions were assessed using specific antibodies: anti-ADP-glucose pyrophosphorylase (AGPase) for the stroma (ap Rees, 1995), anti-Tic40 for the envelope, and anti-LHC chlorophyll a/b binding protein 1 (Lhcb1) for the thylakoid membrane (Farmaki *et al.*, 2007). AQP1 was detected in the envelope and thylakoid membrane fractions but no signal was seen for the stroma soluble fraction (Fig. 3). As expected, a stronger signal was detected in the AQP1 and TicAQP1 transplastomic plants than in the WT plants. Monomeric and oligomeric forms were detected in the envelope and thylakoid fractions of both types of transplastomic plants. The AQP1 signal was most intense in the envelope fraction; note that 10-fold more thylakoid fraction protein was loaded to enable the detection of AQP1. Monomers and dimers were present mainly in the envelope fraction, with faint signals detected in the thylakoid fraction. Trimers and tetramers were detected in the envelope fraction but not in the thylakoid fraction. A higher proportion of high molecular weight aggregates was observed in the thylakoid fraction of TicAPQ1 plants relative to the envelope fraction. Immunoblotting could not provide an accurate estimate of the relative distribution of NtAQP1 in each membrane fraction owing to the disproportionate method by which each membrane fraction was purified. However, the higher *NtAQP1* expression level in TicAQP1 transplastomic plants correlated with the higher AQP1 content in the thylakoid and envelope membranes of these plants relative to AQP1 transplastomic plants (Fig. 3).

### Photosynthetic performance and protein and starch metabolism were impaired by chloroplast NtAQP1 overexpression

The transplastomic plants, particularly TicAQP1 plants, showed retarded growth in comparison to the WT plants during the first 3 weeks following transplantation into pots, but thereafter they caught up and reached a similar height under standard growth conditions (data not shown).

**Fig. 4.**
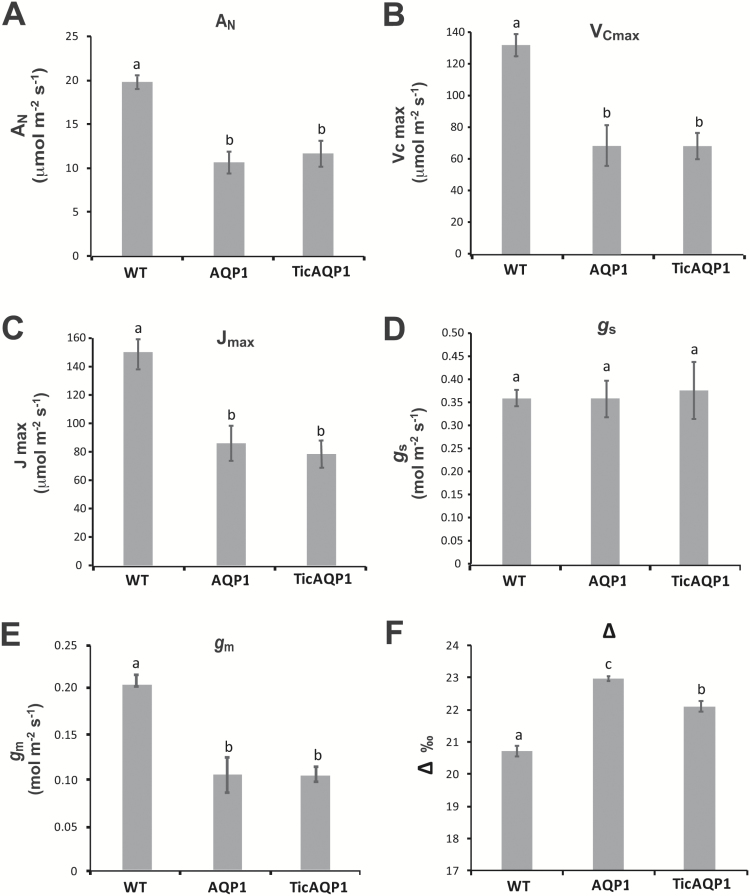
(A) Net photosynthesis, (A_N_), (B) maximum carboxylation velocity of Rubisco (*V*_Cmax_), (C) maximum electron transport rate contributing to ribulose 1,5-bisphosphate regeneration (*J*_max_), (D) stomatal conductance (*g*_*s*_), (E) mesophyll conductance (*g*_*m*_), and (F) ^13^C isotope discrimination (Δ) of wild-type (WT) and AQP1 and TicAQP1 transplastomic plants. Representative data from two independent experiments are presented. Values are means ±SE (*n*=7). Different letters indicate significantly different values (ANOVA, *P*<0.05).

**Fig. 5.**
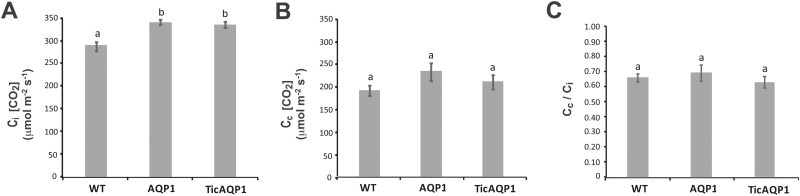
(A) Intercellular CO_2_ concentration (C_i_), (B) chloroplast CO_2_ concentration (C_c_), (C) and C_c_/C_i_ ratio of wild-type (WT) and AQP1 and TicAQP1 transplastomic plants. Representative data from two independent experiments are presented. Values are means ±SE (*n*=7). Different letters indicate statistically different values (ANOVA, *P*<0.05).

Net photosynthesis (A_N_) analyses showed both the AQP1 and the TicAQP1 plants to have lower CO_2_ fixation rates than the WT plants (Fig. 4A), with no differences between the transplastomic plants. As expected, no significant differences were observed between genotypes for leaf stomatal conductance (*g*_s_) (Fig. 4D). In contrast, *g*_m_ was diminished by 50% in AQP1 and TicAQP1 plants relative to WT plants (Fig. 4E). A_N_/C_i_ curve determinations highlighted that the Rubisco maximum carboxylation capacity (*V*_Cmax_) and the maximum electron transport rate contributing to ribulose 1,5-bisphosphate regeneration (*J*_max_) values measured in both transplastomic plants were lower than those in WT plants (Fig. 4B, C). Gas exchange analyses also showed that the transplastomic plants had a higher C_i_ value (Fig. 5A), while no significant differences in C_c_ were detected (Fig. 5B). The C_c_/C_i_ ratio was not altered in the transplastomic plants relative to the WT plants (Fig. 5C). In contrast, compared with the WT plants, AQP1 and TicAQP1 plants showed higher ^13^CO_2_ discrimination (Δ) values (Fig. 4F). Transplastomic plants had lower soluble protein levels than their WT counterparts (Table 1). Moreover, the Rubisco levels were strongly reduced in the AQP1 and TicAQP1 plants (reductions of 23% and 41%, respectively). A 4-fold reduction in the leaf starch content, and reduced chlorophyll levels, were detected in the transplastomic plants relative to the WT plants (Table 1). In addition, the leaf mass area was reduced in the transplastomic plants (12.5% in AQP1 and 15.9% in TicAQP1 plants).

**Table 1.** Biochemical variables measured in young leaves from wild-type (WT) and transplastomic plants grown in a growth chamber

	WT	AQ1	TicAQP1
Starch (μmol Glu g FW^−1^)	21.7 ± 2.9^**a**^	5.5 ± 0.2^**b**^	5.7 ± 0.4^**b**^
Soluble protein (mg g FW^−1^)	16.1 ± 0.7^**a**^	13.3 ± 1.0^**ab**^	12.3 ± 0.4^**b**^
Insoluble protein (mg g FW^−1^)	13.0 ± 1.7^**a**^	10.5 ± 1.3^**a**^	10.2 ± 2.4^**a**^
Rubisco (% relative to WT)	100 ± 3.4^**a**^	76.7 ± 3.9^**b**^	58.8 ± 5.4^**c**^
Chlorophyll content (SPAD)	42.9 ± 1.0^**a**^	34.2 ± 0.9^**b**^	30.0 ± 0.6^**c**^

Values are means±SE (*n*=5–7).

Different superscript letters denote significant differences (ANOVA, *P*<0.05).

The chlorophyll content was measured using a Minolta SPAD 502 chlorophyll meter.

**Fig. 6.**
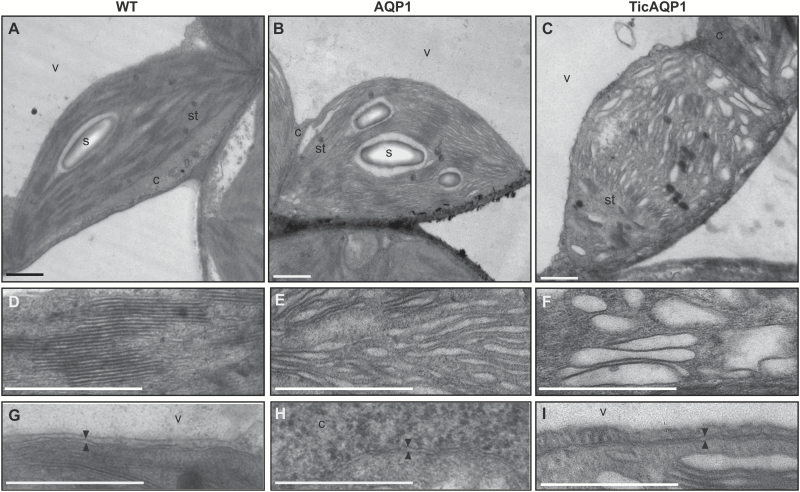
Changes in chloroplast ultrastructure due to *AQP1* overexpression from the plastid genome. Transmission electron microscopic images of chloroplasts from (A) wild-type (WT), (B) AQP1, and (C) TicAQP1 plants. (D–F) Detail of the thylakoids from (D) WT, (E) AQP1, and (F) TicAQP1 plants. (G–I) Detail of the chloroplast envelope (delineated by two arrowheads) in (G) WT, (H) AQP1, and (I) TicAQP1 plants. C, cytosol; s, starch granule; st, stroma; v, vacuole. Scale bar=1 μm.

The ultrastructure of the mesophyll cell chloroplasts was analysed by transmission electron microscopy. Major differences were observed between the WT and transplastomic plants (Fig. 6). The WT plants showed the normal architecture of the thylakoid network, arranged in grana and lamellae (Fig. 6A), while the AQP1 and TicAQP1 plants showed abnormal granal stacking with a reduced number of appressed thylakoids (Fig. 6B, C). In comparison to the normal granal structure of the WT plants (Fig. 6D), the transplastomic plants (especially the TicAQP1 plants) also showed defective grana and swelling of the thylakoid lumen (Fig. 6E, F). Large protein aggregates detected by western blot (Figs 2B and 3), particularly in TicAQP1 plants, could have affected the thylakoid membrane integrity, resulting in abnormal thylakoid architecture. No differences were observed in the envelope membranes (Fig. 6G–I).

The integrity of the thylakoid membranes was assessed by treating isolated chloroplasts with SDS, a product commonly used in cell permeation assays in bacteria (Griffith and Wolf, 2002). For the chloroplasts of the AQP1 and especially the TicAQP1 plants, total chlorophyll solubilization was obtained with lower SDS concentrations than those required to achieve the same effect with WT plant chloroplasts (Supplementary Fig. S2A, C). The same pattern was observed for the solubilization of AQP1 and the thylakoid membrane Lhcb1 proteins, but not for the stromal AGPase or thylakoid-lumen-associated TL29 proteins (Supplementary Fig. S2B).

## Discussion

### Targeting of recombinant AQP1 to chloroplast membranes

The present study shows that NtAQP1 can be overexpressed from the plastid genome and that it localizes to the chloroplast membranes. Native NtAQP1 localizes to both the plasma membrane and the chloroplast IEM. No transit peptide for its chloroplast targeting has been identified, and the sorting mechanisms responsible for this dual localization are unknown (Luu and Maurel, 2013). The expression of *NtAQP1* from the plastid genome results in the AQP1 polypeptide being synthesized in the stroma and not in the cytosol as in WT plants, conditioning its subsequent import pathway to the IEM. Hence, in addition to the *AQP1* coding sequence, and given the uncertainty that the recombinant AQP1 from the chloroplast stroma would reach its target location correctly, a second construct with the transit peptide of the IEM Tic40 protein fused to AQP1 was prepared for plastid transformation. Tic40 is an integral IEM protein involved in protein translocation across this membrane (Chou *et al.*, 2003) that follows the post-import pathway for IEM targeting (Li and Schnell, 2006). Both constructs resulted in the incorporation of AQP1 in the chloroplast envelope and the thylakoid membranes. This result shows that the topology-determining sequence information within NtAQP1 is sufficient for its integration into the envelope membranes from the stroma (Fig. 3). Chloroplast IEM proteins that follow the post-import pathway are first imported from the cytoplasm into the chloroplast stroma in the form of a soluble, processed, intermediate product, and subsequently reinserted from the stroma into the IEM. NtAQP1 expressed from the plastid genome could putatively use the second part of this pathway from the stroma to the IEM. It has been shown that conserved proline residues in the N-terminal region of IEM proteins such as Tic40 and Tic110 are required for stromal reinsertion (Chiu and Li, 2008). The six proline residues present at positions 21, 35, 36, 37, 39, and 43 of NtAQP1 suggest that it uses a common import mechanism from the stroma.

Native IEM proteins utilize two different pathways for their targeting. It is unknown whether the native AQP1, with six membrane-spanning alpha helices, uses the post-import pathway or the stop-transfer pathway. Very little is known about the insertion of polytopic IEM proteins. For instance, the Cor413im1 membrane protein, with five or six transmembrane domains, is incorporated in the IEM via the stop-transfer pathway (Okawa *et al.*, 2014), while Tic110, which has six transmembrane domains, utilizes the post-import pathway (Lübeck *et al.*, 1997; Li and Schnell, 2006). If native and plastidial AQP1 use different import pathways, this could result in an improper location within the IEM that negatively affects its functionality. Tic40, a component of the TIC complex, is involved in the reinsertion process for proteins that use the post-import pathway (Chiu and Li, 2008). It could be expected that the recombinant TicAQP1 protein, which includes the Tic40 transit peptide, is inserted close to and potentially interacts with the TIC complex. It remains to be elucidated whether the IEM import pathways of recombinant TicAQP1 and AQP1 proteins are the same, but the physiological performance of both transplastomic plants was similar. The putative drawback related to IEM import seems to be equivalent in both transplastomic plants, irrespective of the presence of the Tic40 transit peptide.

However, integration of NtAQP1 into the thylakoid membranes was unexpected, and the mechanism of protein sorting remains unknown. Plastid transformation has also allowed the successful integration of other foreign proteins, with or without signal peptides, to the thylakoid membranes (Henig *et al.*, 2007; De Marchis *et al.*, 2011; Ahmad *et al.*, 2012; Shanmugabalaji *et al.*, 2013; Scotti *et al.*, 2015), indicating that different import mechanisms might be used. The expression of the *Synechococcus* BicA bicarbonate transporter in tobacco plastids unexpectedly resulted in dual targeting of the protein to the thylakoid membranes and, in a smaller proportion, to the chloroplast envelope (Pengelly *et al.*, 2014). A model for contact zones between plasma and thylakoid membranes, allowing protein trafficking in short-lived connection assemblies, has been proposed for the cyanobacterium *Synechocystis* (Pisareva *et al.*, 2011). In addition, functional thylakoid membranes were developed in association with the chloroplast envelope in *Chlamydomonas* under certain conditions (Hoober *et al.*, 1991). These and other investigations have suggested a role of the IEM for thylakoid biogenesis in vascular plants (Celedon and Cline, 2013). This mechanism might tentatively explain the dual targeting of recombinant AQP1 to the envelope and thylakoid membranes.

### Physiological performance of transplastomic plants

The present study sought to determine whether CO_2_ transport to the chloroplast could be boosted by increasing the amount of AQP1 in the chloroplast membranes. Much higher AQP1 protein levels (up to 16-fold higher than in the WT) were obtained in this study than in another study using nuclear transformation, in which double the levels in WT were obtained (Flexas *et al.*, 2006); this difference was probably due to the plastid transformation method. Despite the integration of AQP1 into the chloroplast envelope membranes, the transplastomic plants overexpressing *NtAQP1* showed lower photosynthetic rates than the WT plants. Associated with the low values of A_N_, transplastomic plants showed both a reduction in CO_2_ diffusion capacity (associated with *g*_*m*_ but not *g*_*s*_) and a lower photosynthetic capacity (*V*_Cmax_ and *J*_max_). Because the different lines differed in their C_i_, and *g*_*m*_ responds to C_i_ (Flexas *et al.*, 2007*a*), the observed differences in *g*_*m*_ could be attributable to differences in C_i_. However, *g*_*m*_/C_i_ ratios (obtained from data shown in Figs 4E and 5A) were 0.3 × 10^–3^ for the two transplastomic lines, compared with ~0.7 × 10^–3^ for the WT, suggesting that *g*_*m*_ is reduced in the transplastomic lines regardless of their C_i_. Considering that between 200 and 400 μmol CO_2_ mol^−1^ air *g*_*m*_ decreases at an approximate rate of 0.1% per μmol CO_2_ mol^−1^ air (shown for different species, including tobacco, by Flexas *et al.*, 2008), if WT plants had had the same C_i_ as the transplastomic plants, their *g*_*m*_ would have decreased by ~5%, that is, it would have still been significantly higher.

The realized photosynthesis achieved by a plant can be manipulated by two means (Gago *et al.*, 2014; Flexas *et al.*, 2016): either by modifying the photosynthetic capacity of the plant (i.e. changing the rate of photosynthesis for a given substrate availability) or by changing the diffusion capacity of the leaf (which, in turn, will modify the quantity of substrate available for photosynthesis). In this study, increasing AQP1 expression dramatically decreased the photosynthesis of the transplastomic plants. The reduced A_N_ was an unexpected result, especially given the role of AQP1 in CO_2_ diffusion, as described in tobacco (Uehlein *et al.*, 2003; Flexas *et al.*, 2006) and in other species (Hanba *et al.*, 2004; Sade *et al.*, 2010). In a previous study, Flexas *et al.* (2006) showed that, compared with the corresponding WT plants, photosynthetic rates were lower in NtAQP1-deficient plants and higher in NtAQP1-overexpressing plants, suggesting that variations in the photosynthetic rate were certainly linked to changes in C_c_ (Flexas *et al.*, 2006). In the present study, the higher C_i_ detected in the transplastomic (compared with the WT) plants indicates that stomatal opening was not involved in the lower A_N_ of the AQP1 and TicAQP1 plants. Indeed, the same stomatal conductance in the three genotypes, along with a clearly lower CO_2_ fixation by the photosynthetic machinery in the transplastomic plants, might be related to the higher C_i_ in AQP1 and TicAQP1 plants. Following from this observation, it could be tentatively concluded that the lower A_N_ in the transplastomic plants was caused by their lower *g*_*m*_ (by ~50%) compared with the WT plants, since *g*_m_ is now recognized to play a major role in CO_2_ diffusion into the chloroplast (Flexas *et al.*, 2006; Flexas *et al.*, 2007*b*; Scafaro *et al.*, 2011; Kaldenhoff, 2012; Evans and von Caemmerer, 2013; Flexas *et al.*, 2013). Nevertheless, the hypothesis on limited diffusion (reduced *g*_*m*_) in AQP1 and TicAQP1 plants cannot explain alone their lower photosynthetic rates. In fact, C_c_ was not different between WT and transplastomic plants, and nor was the C_c_/C_i_ ratio, despite very different rates of photosynthesis (Figs 4 and 5). Although *g*_*s*_ was not affected by the overexpression of AQP1 in transplastomic plants, the overall CO_2_ supply was reduced as a consequence of the reduction of *g*_*m*_. However, because the CO_2_ demand was also reduced due to impaired *V*_Cmax_ and *J*_max_, the overall result was a relatively higher C_c_ in the transplastomic plants compared with WT plants; the difference was more evident according to the Δ measurements (Fig. 4F) than according to chlorophyll fluorescence-based estimates (Fig. 5B). If C_c_ (i.e. the substrate for photosynthesis) was the same between the WT, AQP1, and TicAQP1 plants, then differences in A_N_ were more likely related to differences in photosynthetic capacity.

Evidence that reduced *V*_Cmax_ and *J*_max_ are the true factors responsible for decreased A_N_ in the two transplastomic lines arises from reverse photosynthesis modelling and from carbon isotope discrimination. Using reverse modelling, it is possible to estimate how large C_c_ and *g*_*m*_ should be for the transplastomic lines to reach WT A_N_ if their *V*_Cmax_ and *J*_max_ is reduced. It turns out from this simulation that C_c_ should increase from the estimated ~230 μmol mol^−1^ to ~330 μmol mol^−1^, which would require *g*_*m*_ values of 8.8 and 4.3 μmol CO_2_ m^−2^ s^−1^ for AQP1 and TicAQP1, respectively; these are unrealistically large values, far out of the range of estimates for any plant species (Flexas *et al.*, 2012). On the other hand, stable isotopes, such as δ^13^C, have been proposed as indicators of stomatal opening and CO_2_ diffusion (Farquhar *et al.*, 1989; Araus *et al.*, 2003). Changes in Δ have been linked to changes in CO_2_ availability and/or Rubisco carboxylation activity (Farquhar *et al.* 1989; Brugnoli and Farquhar, 2000; Ghashghaie *et al.*, 2003). The fact that CO_2_ supply was reduced in transplastomic plants due to the reduction of *g*_*m*_, but Δ was still larger in these plants than in WT plants, suggests a reduction of carboxylase activity relative to CO_2_ supply around Rubisco.

The localization of AQP1 to the thylakoid membranes might negatively affect their functionality, perhaps via interaction with proteins of the photosynthetic apparatus that affects the protein dynamics within the thylakoid, with a consequent impact on photosynthetic capacity. Indeed, the reduced membrane resistance to SDS (Supplementary Fig. S2) suggests that the thylakoid membrane was damaged in some way, possibly related to the presence of recombinant AQP1. It has been reported that targeting foreign membrane proteins to thylakoid membranes by chloroplast transformation causes mutant phenotypes with reduced growth and photosynthetic capacity, altered thylakoid ultrastructure, and impairment of the integrity of the photosystems (Henig *et al.*, 2007; Gnanasekaran *et al.*, 2016). A similar response could be produced by the recombinant AQP1 located in the thylakoid membranes, particularly considering the presence of large protein aggregates (Fig. 3). However, despite the higher content of recombinant AQP1 in the thylakoid membranes of TicAQP1 plants, the photosynthetic parameters were similar in both AQP1 and TicAQP1 plants (Fig. 4). Perhaps a threshold of chloroplast damage was reached in the AQP1 plants and the greater content of the recombinant protein in TicAQP1 plants was inconsequential. Another possibility is that other processes, in addition to the functionality of the thylakoids, might be involved in the photosynthetic impairment of these plants.

### Other factors affecting recombinant AQP1 functionality

The gas exchange data highlight the fact that, in contrast to what was expected, *NtAQP1* overexpression from the chloroplast genome constrains CO_2_ diffusion from the substomatal cavity to the chloroplast. An explanation for this harmful effect is not easy to provide, but other factors, in addition to the above-mentioned factors related to the import pathway and thylakoid targeting, could be involved.

The regulation of the function of AQP proteins depends on several processes, including post-translational modifications and protein interactions, that affect both their activity and their subcellular localization (Hove and Bhave, 2011; Chaumont and Tyerman, 2014; Verdoucq *et al.*, 2014). It is possible that differences between the stromal and cytosolic environments prevent the required AQP post-translational modifications and/or interactions in the stroma.

Another important aspect to be considered is that AQPs assemble as homo- and/or heterotetramers in the membranes. The AQP monomer is the functional unit for water transport, but the tetramer and its composition may be important for CO_2_-related transport. Further, in tobacco, CO_2_ diffusion is greater when tetramers consist only of NtAQP1 from the PIP1 family (Otto *et al.*, 2010), and different proportions of PIP1 and PIP2 subunits in the tetramer may promote either water or CO_2_ transfer, or both (Flexas *et al.*, 2012). If improper AQP1 monomers (due to incorrect post-translational modifications) synthesized in the chloroplast stroma interact with normal WT AQP1 synthesized in the cytosol, non-functional or partially functional homotetramers might find their way to the IEM. It must be noted that unusual oligomeric and possible non-specific protein aggregates were detected (Figs 2B and 3). Given that the transplastomic plants in this study had 10–16 times the AQP1 content of the WT plants, defective homotetramers might have prevailed over any homotetramers composed exclusively of WT AQP1 monomers, thus compromising AQP1-mediated CO_2_ transport in the IEM.

Finally, despite the engineered modification to AQP1 expression levels being able to alter *g*_m_ (suggesting a role for AQPs in CO_2_ transport), the molecular bases controlling *g*_m_ remain unclear and are affected by components such as the cell walls, plasma and chloroplastic membranes, and carbonic anhydrases. Thus, other regulatory mechanisms that control *g*_m_ might be unmasked in plants with altered levels of AQPs (Verdoucq *et al.*, 2014), such as those analysed in the present study.

In conclusion, in an attempt to increase CO_2_ diffusion across the chloroplast membranes we engineered tobacco plants with substantially increased levels of the CO_2_-permeable NtAQP1 protein within the chloroplast membranes. However, there was no improvement in intracellular CO_2_ transfer capacity in the transplastomic plants. In fact, we observed impairment in photosynthetic capacity, which could be partially attributed to changes in the thylakoid ultrastructure, and reductions in *V*_Cmax_ and *J*_max_, which were the main limiting factors. This study serves to highlight obstacles that need to be overcome in work towards engineering improved CO_2_ transfer capacity for enhanced photosynthesis.

## Supplementary data

Supplementary data are available at *JXB* online.

Fig. S1. Relationship between ΦPSII versus ΦCO_2_ used to correct ETR.

Fig. S2. Membrane disruption of chloroplasts from wild-type (WT), AQP1, and TicAQP1 plants with increasing concentrations of SDS.

Supplementary Material
