# Supplementary material for: Physiological performance of transplastomic tobacco plants overexpressing aquaporin AQP1 in chloroplast membranes
Source: J Exp Bot. 2018 Apr 18;69(15):3661–73. doi: 10.1093/jxb/ery148 (PMC6022695; doi:10.1093/jxb/ery148)
Supplement: Supplementary Material [file erx148_suppl_supplementary-material.pdf]

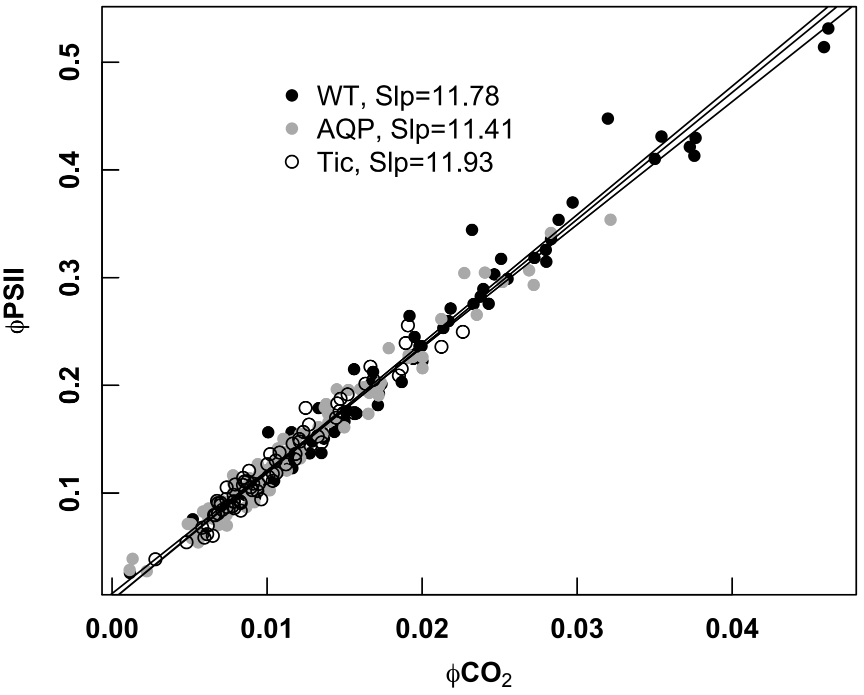

Supplementary Figure S1. Relationship between ΦPSII *vs* ΦCO_2 used to correct ETR. Values of the slope for each genotype are indicated in the legend. The α*β factor was calculated as 4/slope, for each single plant measured (see M&M, Valentini *et al.*, 1995 ; Pons *et al.*, 2009 and Martins *et al.,* 2013).

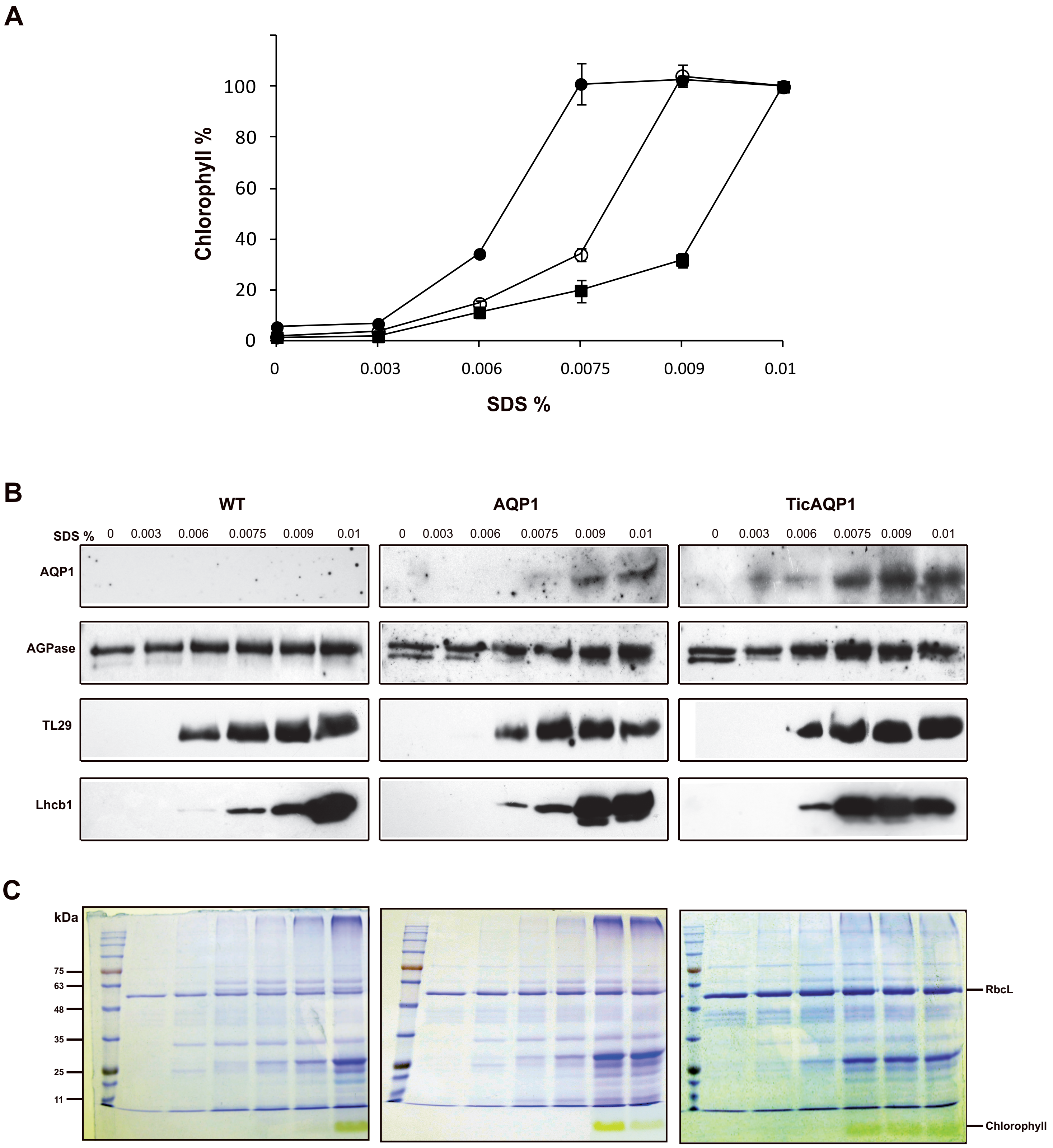

Supplementary Figure S2. Membrane disruption of wild-type (WT), AQP1 and TicAQP1 chloroplasts treated with increasing concentrations of SDS. (A) Chlorophyll released after a 10 min treatment with the corresponding SDS concentration. WT (square), AQP1 (open circle) and TicAQP1 (filled circle) (n=3). (B) Immunoblot analysis with antibodies against AQP1, AGPase, TL29 (thylakoid lumen protein) and Lhcb1 (thylakoid membrane protein) of supernatants after each treatment. (C) Coomassie blue-stained SDS-PAGE of supernatants from each fraction.
